# New Drugs, Appliances, and Things Medical

**Published:** 1892-06-25

**Authors:** 


### NEW DRUGS, APPLIANCES, AND THINGS MEDICAL.

[All preparations, appliances, novelties, etc., of which a notice is desired, should be sent for The Editor, to care of The Manager, 140, Strand, London, W.C.]

### WYETH'S BEEF JUICE.

(Agents, Roberts and Co., New Bond Street.)

This is a well-made beef juice with a very high percentage of soluble coagulable albumen. On heating a portion in a test-tube, the whole becomes so solid that the tube can be inverted without spilling a drop. The flavour is also pleasant. It has not the mawkish insipidity of some similar preparations, but has that peculiarly grateful taste that well prepared meat carries with it. So well made is it that the hæmoglobin of the meat is unaltered and can be recognised by its bands in the spectroscopic field, and by the change in colour and precipitation with coagulum in boiling. It can be taken in any of the usual ways, such as in iced water or soda water. Never mix with boiling water, or the coagulation of the albumen resulting deprives it of its "beef juice" properties, though the same mixture makes, with the usual flavouring, a most excellent beef-tea. Among the many candidates for favour in the beef-juice class, we know of none better than Wyeth's.

### THE ANTI-MEDICINE TASTER.

(The Druggists' Speciality Co., 44, Stockorchard Road, N.)

This is a simple contrivance for taking disagreeable or acid medicines, or for administering liquid food. It consists of a glass tube seven to eight inches long, one end of which is bulbous, with an aperture in one side for the forefinger; the other end is slightly curved. To use it, slightly incline the tube, and fill through the side aperture. Place the forefinger lightly on the aperture, to prevent the escape of fluid from the tube. Pass the narrow end along the roof of the mouth (not along the tongue) as far down the throat as possible, raise the forefinger from the aperture and the fluid will flow down the throat. By replacing the finger on the aperture the flow of the liquid can be instantly stopped. We have no doubt that in many instances this little apparatus will be of service to those who are obliged to take unpleasant or acid medicine, and so fear the effect on their teeth, and also for the feeding of the sick who are so feeble as not to be able to drink in the ordinary way. The price is one shilling.

### BRYONIA.

Dr. Gerard Smith writes objecting to our description of Bryonia as a "new remedy." It has, he states, been in use for sixty years, which is, no doubt, the fact, though that is a short period in art. But we would suggest for Dr. Gerard Smith's consideration that "new" and "old" are relative terms; that, though Bryonia has been "in use,' it has not been in "general use"; that if it be good, as it is, its value ought to be made more widely known; and that it makes little matter to those who are not familiar with it whether it be called a "new," a "middle-aged," or an "old" remedy. It is, at any rate, "new" to them. If Dr. Smith wishes to claim the discovery of Bryonia for homœopathy, let him do so boldly. But that is quite another question.

